# Genome‐wide association study of metabolites in patients with coronary artery disease identified novel metabolite quantitative trait loci

**DOI:** 10.1002/ctm2.290

**Published:** 2021-01-27

**Authors:** Zixian Wang, Qian Zhu, Yibin Liu, Shiyu Chen, Ying Zhang, Qilin Ma, Xiaoping Chen, Chen Liu, Heping Lei, Hui Chen, Jing Wang, Shufen Zheng, Zehua Li, Lingjuan Xiong, Weihua Lai, Shilong Zhong

**Affiliations:** ^1^ Department of Pharmacy Guangdong Provincial People's Hospital, Guangdong Academy of Medical Sciences Guangzhou China; ^2^ Guangdong Provincial Key Laboratory of Coronary Heart Disease Prevention, Guangdong Cardiovascular Institute, Guangdong Provincial People's Hospital Guangdong Academy of Medical Sciences Guangzhou China; ^3^ School of Biology and Biological Engineering South China University of Technology Guangzhou China; ^4^ School of Pharmaceutical Sciences Southern Medical University Guangzhou China; ^5^ Department of Cardiology, Guangdong Provincial People's Hospital Guangdong Academy of Medical Sciences Guangzhou China; ^6^ Department of Cardiology, Xiangya Hospital Central South University Changsha China; ^7^ Department of Clinical Pharmacology, Xiangya Hospital Central South University Changsha China; ^8^ Department of Cardiology the First Affiliated Hospital of Sun Yat‐sen University Guangzhou China

Dear Editor,

We performed a metabolome‐based genome‐wide association study (metaboGWAS) in a Chinese cohort with coronary artery disease (CAD). We identified seven novel metabolite quantitative trait loci (metaboQTLs) and revealed the possible clinical significance of these metaboQTLs. Our study may add novel biological insights into the metabolic background of disease‐related variants and contribute to the development of risk prediction and treatment of CAD.

CAD is the most common type of cardiovascular disease worldwide, with high morbidity and mortality.^1^ To date, genetic variants can explain approximately 10.6% of CAD heritability,^2^ whereas the internal biological information is often limited. Plasma metabolites represent a functional intermediate for genetic makeup and environmental exposure to the end phenotype that can be used to uniquely identify individuals.^3^ Understanding the genetic regulation of the levels of circulating metabolites in CAD can illustrate pathophysiological signals and guide the tool development for risk prediction and therapies. metaboGWAS is an effective way to discover important metaboQTLs,^4^ which can provide deep insights into the biological links and molecular mechanisms between genes and metabolites. To date, hundreds of metaboQTLs have been uncovered.^5^, ^6^, ^7^ The reported metaboQTLs exhibit greater effect size and stronger association with metabolic traits than with complex traits. However, most metaboGWASs were conducted on European populations, and a limited number were performed on Chinese populations, especially in CAD cohorts.

Here, we present the first large‐scale metaboGWAS of a Han Chinese population to identify effective metaboQTLs. Figure 1 depicts the study flow, and Figure S1 presents an overview of the patients’ enrollment. This work is a two‐stage study and involved 1551 subjects with CAD from three clinical centers (Supporting Information Methods). Table 1 provides the patient characteristics. All patients diagnosed with CAD via angiography had undergone percutaneous coronary intervention, and the exclusion criteria were the same as our previous study.^8^ Plasma samples from the patients with CAD were profiled for metabolites by using widely targeted metabolomics, and DNA was collected from hemocytes for genotyping (Supporting Information Methods). Spearman correlation was used to analyze the pairwise correlation between these 161 metabolites in the discovery group (Tables S1 and S2). Table S3 summarizes the data of the two groups.

**FIGURE 1:**
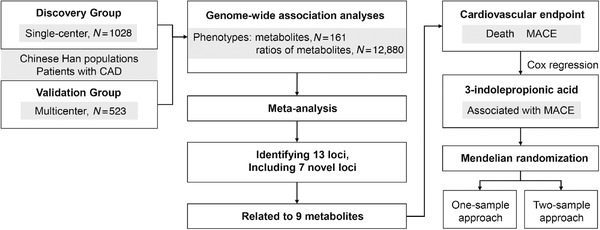
Schematic of the study design for primary analysis. Abbreviations: CAD, coronary artery disease; MACE, major adverse cardiovascular events

**TABLE 1:** Baseline characteristics of the two groups

	Value *N* (%) or mean ± SD		
Characteristics	Discovery group	Validation group	*p*
**Demographic data**			
Size	1028	523	–
Age	62.98 ± 10.05	61.94 ± 10.14	0.0474
Sex (male)	818 (79.57)	382 (73.04)	0.0028
BMI (kg/m^2^)	24.27 ± 4.80	24.10 ± 3.07	0.7523
**Comorbidities**			
Arrhythmia	92 (8.97)	47 (8.99)	0.9852
Diabetes	281 (27.39)	151 (28.87)	0.5331
Heart failure	89 (8.67)	241 (46.08)	<0.0001
Hypertension	618 (60.18)	315 (60.23)	0.9574
Hyperlipidemia	119 (11.59)	71 (13.58)	0.2947
**Baseline biochemical measurements**			
ALT (U/L)	27.43 ± 13.19	26.80 ± 23.06	0.5960
AST (U/L)	26.65 ± 10.65	26.40 ± 38.37	0.8622
CK (U/L)	111.47 ± 111.49	124.97 ± 348.94	0.4652
eGFR (ml/min/1.73 m^2^)	94.58 ± 74.06	94.58 ± 122.97	0.9993
CKMB (U/L)	7.48 ± 5.94	13.37 ± 17.55	<0.0001
CHOL (mmol/L)	4.28 ± 1.13	4.30 ± 1.79	0.7706
LDLC (mmol/L)	2.58 ± 0.93	2.72 ± 1.00	0.0103
HDLC (mmol/L)	0.97 ± 0.26	1.00 ± 0.25	0.0233
TRIG (mmol/L)	1.61 ± 1.12	1.83 ± 1.87	0.0184
GLUC (mmol/L)	6.71 ± 2.69	5.96 ± 2.20	<0.0001
Lpa (mg/L)	307.16 ± 329.42	284.55 ± 330.01	0.3153
APOA (g/L)	1.04 ± 0.28	1.17 ± 0.24	<0.0001
BNP (pg/ml)	777.40 ± 1604.48	1351.12 ± 3869.26	0.3297
**Medication**			
β‐blockers	917 (89.38)	447 (85.47)	0.0314
ACEIs	653 (63.65)	253 (48.37)	<0.0001
CCBs	292 (28.46)	158 (30.21)	0.5192
PPIs	293 (48.44)	353 (67.50)	<0.0001
SYNTAX	16.44 ± 10.75	15.88 ± 13.33	0.8835

Abbreviations: ACEIs, angiotensin converting enzyme inhibitors; ALT, alanine aminotransferase; APOA, apolipoprotein a; AST, aspartate aminotransferase; BMI, body mass index; BNP, B‐type natriuretic peptide; CCBs, calcium channel blockers; CHOL, cholesterol; CK, creatine kinase; CKMB, creatine kinase MB; eGFR, estimated glomerular filtration rate; HDLC, high‐density lipoprotein cholesterol; GLUC, glucose; Lpa, lipoprotein (a); LDLC, low‐density lipoprotein cholesterol; PPIs, proton pump inhibitors; SYNTAX score, Synergy between PCI with TAXUS and Cardiac Surgery score; TRIG, triglyceride.

Primary association analysis (Supporting Information Methods) was conducted using 3,435,396 imputed single‐nucleotide polymorphisms (SNPs) with 161 metabolites and 12,280 metabolite ratios in two independent groups. The first 10 principal components, sex, age, aspartate aminotransferase (AST), antihypertensive drugs, hypertension, estimated glomerular filtration rate (eGFR), and diabetes were included as covariates in both groups. We used meta‐analysis to combine the association results of the two groups. P‐gain was used as a standard to integrate the results of association analysis between single metabolites and ratios.^9^ Bonferroni *p* < 5 × 10^−8^ / 161 = 3.11 × 10^−10^ and *p* < 5 × 10^−8^ / 12,880 = 3.88 × 10^−12^ were used as thresholds for significant single metabolite and metabolite ratio associations, respectively. After heterogeneity test, P‐gain, and linkage disequilibrium (LD) analyses, the numbers of these associations collapsed to 109, including 13 metabolite and 96 ratio associations, and all of them contained 15 SNPs (Table S4). One SNP (rs73530508) was removed after LD analyses of the merged data. The association of each locus with the lowest *p*‐value against any metabolites and ratios was reported (Figure 2A and B and Supporting Information Figures S2–S12). We searched the GWAS Catalog^10^ (last data release on August 13, 2020) for the reported or possibly novel loci. We identified 13 loci (Table 2), including seven novel loci, namely, *RHPN1* with the ratio of L‐histidine and uric acid, *ZBTB16* with inosine, *LOC112268121* with eudesmic acid, *SLC10A1* with glycocholic acid, *GABRG3* with dodecanedioic acid, *ACSM2B* with 3‐indolepropionic acid (IPA), and *PWP2* with 1,4‐dihydro‐1‐methyl‐4‐oxo‐3‐pyridinecarboxamide.

**FIGURE 2:**
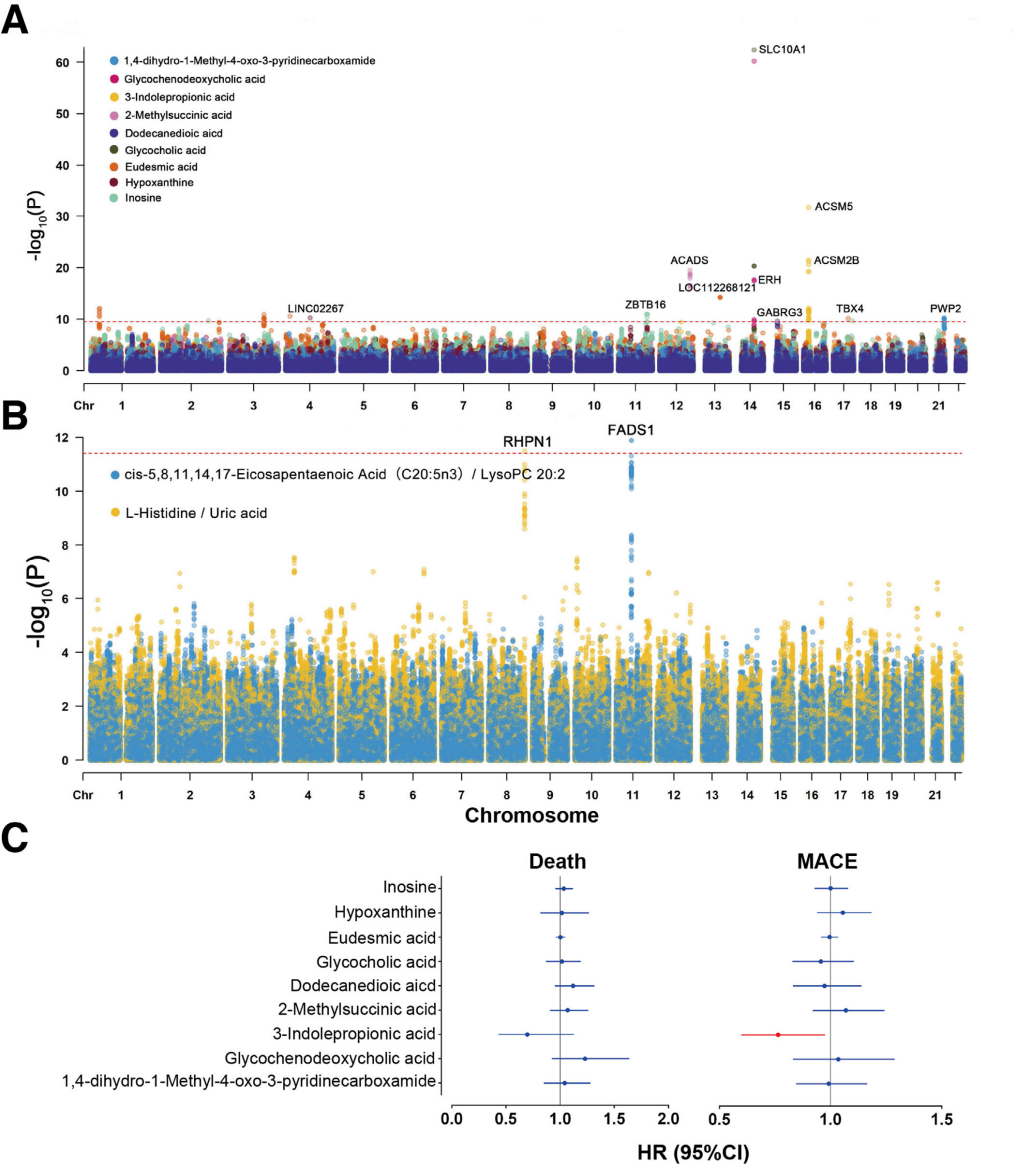
Plots for loci with metabolites/ratios and relationship between metaboQTL‐related metabolites and risk of death and MACE. (A and B) Manhattan plots for the loci in meta‐analysis. (A) Red dashed line indicates the threshold of genome‐wide significance on single metabolites (*p* < 3.11 × 10^−10^). (B) Red dashed line indicates the threshold of genome‐wide significance of metabolite ratios (*p* < 3.88 × 10^−12^). (C) Forest plots of the HR of death and MACE of metaboQTL‐related metabolites in the discovery group. The effect sizes with 95% CI were plotted by adjusting the covariant in Cox regression model, and the significant metabolites are highlighted in red

**TABLE 2:** Genomic loci associated with metabolites and metabolite ratios at genome‐wide significance in meta‐analysis

Locus	rsID	Metabolite/ratio^a^	Annotation^b^	Ref^c^	*N*	EA/OA^d^	Beta (SE^e^)	*p* ^f^
*LINC02267*	rs117445861	Hypoxanthine	RINT	NAS	1551	A/G	−0.54 (0.08)	5.9E‐11
*RHPN1*	rs73365860	L‐Histidine/uric acid	DS	NL	1513	T/C	−0.17 (0.02)	3.3E‐12
*FADS1*	rs174548	cis‐5,8,11,14,17‐Eicosapentaenoic acid (C20:5n3)/LysoPC 20:2	INT	NAS	1551	C/G	−0.12 (0.02)	1.3E‐12
*ZBTB16*	rs573195	Inosine	INT	NL	1499	T/G	−0.52 (0.08)	1.2E‐11
*ACADS*	rs9204	2‐Methylsuccinic acid	UTR3	NAS	1551	A/G	0.37 (0.04)	3.4E‐20
*LOC112268121*	rs9530695	Eudesmic acid	IG	NL	1506	C/G	2.50 (0.32)	6.1E‐15
*ERH*	rs76199614	Glycochenodeoxycholic acid	DS	NAS	1515	A/G	0.29 (0.04)	1.2E‐10
*SLC10A1*	rs2296651	Glycocholic acid	MV	NL	1551	A/G	−1.31 (0.08)	4.2E‐63
*GABRG3*	rs58275387	Dodecanedioic aicd	INT	NL	1542	T/C	−0.34 (0.05)	2.6E‐10
*ACSM5*	rs9929808	3‐Indolepropionic acid	INT	NAS	1551	T/C	−0.77 (0.07)	2.1E‐32
	rs6497488	3‐Indolepropionic acid	INT	NAS	1500	T/C	−0.40 (0.06)	5.6E‐11
*ACSM2B*	rs11645661	3‐Indolepropionic acid	UTR3	NL	1548	A/G	−0.69 (0.07)	3.7E‐22
*TBX4*	rs8073361	Eudesmic acid	IG	NAS	1551	A/C	−1.97 (0.30)	8.8E‐11
*PWP2*	rs74716564	1,4‐dihydro‐1‐Methyl‐4‐oxo‐3‐pyridinecarboxamide	INT	NL	1525	A/G	−0.47 (0.07)	6.4E‐11

^a^ The “/” represents a ratio relationship, the front is the numerator, the back is the denominator.

^b^ SNP annotation, UTR3 (3′ Untranslated Region), DS (downstream), IG (intergenic), INT (Intronic), MV (Missense Variant), RINT (ncRNA_intronic).

^c^ Novel Locus (NL) or Novel Association (NAS).

^d^ Effect/other allele.

^e^ Standard error.

^f^ Meta‐analysis *p*‐value.

We further explored the biological functions of the 13 loci. We used several databases to investigate the known biological functions and disease or pharmacological correlation of these loci (Supporting Information Methods, Table S5). We observed four loci, including three novel loci, which encode enzymes or transporters in genes. Moreover, 11 loci are related to diseases, including six novel loci. In addition, the effect on drugs of genes in two loci has been studied previously.

Metabolites may affect the prognosis of CAD, but changes in the concentration of certain metabolites may be a concomitant state. To explore whether or not these metaboQTL‐related metabolites affect the occurrence of end‐point events, we used Mendelian randomization (MR) approaches to explain the causal relationship between them. First, we explored the association between metaboQTL‐related metabolites and death and major adverse cardiovascular events (MACE) in the discovery group through Cox regression model (Table S6), adjusting for sex, age, eGFR, AST, hypertension, diabetes, cardiovascular family history, and smoking history. We observed that IPA may reduce the risk of MACE (HR [95%CI], 0.76 [0.60–0.97]) (Figure 2C) and then used two MR approaches (Supporting Information Methods) to explore their causal relationship. IPA was found related to the risk of MACE in the one‐sample MR analysis (HR [95%CI], 0.70 [0.55–0.89]). However, no strong evidence was found in the two‐sample MR analysis. The effect value of the inverse variance weighted method in the two‐sample MR analysis opposed the expectation, and no similar effect was observed in the four other models (Figure S13 and Tables S7 and S8). A large sample size in the future may provide deep insights into the relationship between IPA and MACE, and further work is needed to uncover the role of IPA in the pathogenesis of the disorder.

In sum, this study represents the first large‐scale metaboGWAS study of Chinese populations. We identified seven novel loci that may be unique to Chinese populations with CAD. Using the one‐sample MR approach, we found that IPA was related to the risk of MACE in patients with CAD. The results strengthen our knowledge of the relationships between genetic variations and individualized metabolism in patients with CAD and potentially facilitate the establishment of personalized explanation or markers for biological differences in disease status.

## CONFLICT OF INTEREST

The authors declare that they have no conflict of interest.

## ETHICS APPROVAL AND CONSENT TO PARTICIPATE

This study was approved by the Medical Ethical Review Committee of Guangdong Provincial People's Hospital (Nos. GDREC2010137 and GDREC2017071H) and conducted according to the Declaration of Helsinki. Informed consent (Nos. 20100910 and 20170211) was obtained from all individual participants included in the study.

## AUTHOR CONTRIBUTIONS

Zixian Wang participated in patient recruitment, performed experiment, analyzed the data, and wrote the original draft of the manuscript. Qian Zhu participated in patient recruitment, performed experiment, supervised the data, and revised manuscript. Yibin Liu performed experiment and analyzed the data. Shiyu Chen analyzed the data. Ying Zhang curated the data. Qilin Ma provided resources. Xiaoping Chen curated the data. Chen Liu curated the data. Heping Lei curated the data. Hui Chen established methodology. Jing Wang performed experiment. Shufen Zheng curated the data. Zehua Li made investigation. Lingjuan Xiong curated the data. Weihua Lai provided resources. Shilong Zhong contributed to the conception of the project, design of the study, provision of resources, analyses of the data, and writing of the manuscript.

## AVAILABILITY OF DATA AND MATERIALS

The datasets used and/or analyzed during the current study are available from the corresponding author on reasonable request.

## Supporting information

Supporting Information

Supporting Information
