# Supplementary material for: Genome‐wide association study of metabolites in patients with coronary artery disease identified novel metabolite quantitative trait loci
Source: Clin Transl Med. 2021 Jan 27;11(2):e290. doi: 10.1002/ctm2.290 (PMC7839954; doi:10.1002/ctm2.290)
Supplement: Supplementary file 2 — Supporting Information [file CTM2-11-e290-s002.docx]

**Supporting information**

**Genome-wide association study of metabolites in patients with** **coronary artery disease identified novel metabolite quantitative trait loci**

Zixian Wang ^1, 2, 3 #^, Qian Zhu ^1, 2, 4 #^, Yibin Liu ^1, 2^, Shiyu Chen ^1, 2, 3^, Ying Zhang ^5^, Qilin Ma ^6^, Xiaoping Chen ^7^, Chen Liu ^8^, Heping Lei ^2^, Hui Chen ^1, 2^, Jing Wang ^1, 2, 3^, Shufen Zheng ^4^, Zehua Li ^1^, Lingjuan Xiong ^1^, Weihua Lai ^1^, Shilong Zhong ^1, 2, 3, 4, 5 *^

**Table of Contents**

**Methods 3**

**Study characteristics** 3

**Sample collection and study endpoints** 4

**Widely targeted metabolomic profiling** 4

**Genome-wide SNP genotyping and quality control (QC)** 8

**Genotype imputation and post-imputation QC** 10

**metaboGWAS and meta-analysis** 10

**Metabolite ratio analyses** 11

**Fine mapping of metaboQTLs** 13

**Biological annotation** 13

**Association between metaboQTL-related metabolites and endpoint events** 14

**MR study** 14

**Supplemental Table Legends** **16**

**Supplemental Figure Legends** **17**

**Supplemental Figures** **19**

**Figure S1. Flow chart of the enrolment of the CAD patients**19

**Figure S2. Q-Q plot of the ratio of L-histidine and uric acid p-values in meta-analysis**20

**Figure S3. Q-Q plot of glycocholic acid p-values in meta-analysis**21

**Figure S4. Q-Q plot of glycochenodeoxycholic acid p-values in meta-analysis**22

**Figure S5. Q-Q plot of inosine p-values in meta-analysis**23

**Figure S6. Q-Q plot of 3-Indolepropionic acid p-values in meta-analysis**24

**Figure S7. Q-Q plot of 2-Methylsuccinic acid p-values in meta-analysis**25

**Figure S8. Q-Q plot of dodecanedioic aicd p-values in meta-analysis**26

**Figure S9. Q-Q plot of the ratio of cis-5,8,11,14,17-Eicosapentaenoic Acid (C20:5n3) and LysoPC 20:2 p-values in meta-analysis**27

**Figure S10. Q-Q plot of eudesmic acid p-values in meta-analysis**28

**Figure S11.** **Q-Q plot of 1,4-dihydro-1-Methyl-4-oxo-3-pyridinecarboxamide p-values in meta-analysis**29

**Figure S12. Q-Q plot of hypoxanthine p-values in meta-analysis**30

**Figure S13. Representative total ion flow diagrams between different QC samples under positive ion mode (a) and negative ion mode (b)**31

**Figure S14. Scatter plot for the genetic associations of 3-indolepropionic acid on the risk of MACE** 32

**Reference** **33**

**Methods**

**Study characteristics**

This research was a two-stage study that included 1,551 Han Chinese subjects with coronary artery disease (CAD). A total of 1,028 patients coming from a single centre (Guangdong Provincial People’s Hospital) were designated as the discovery group (62.98±10.05 years old), and 523 patients from three centres (358 patients from Guangdong Provincial People’s Hospital, 161 patients from Xiangya Hospital, Center South University and 4 patients from the First Affiliated Hospital, Sun Yat-sen University) were assigned as the validation group (61.94±10.14 years old). The severity of CAD, as represented by the Synergy between PCI with TAXUS and Cardiac Surgery score, were 16.44±10.75 and 15.88±13.33 for the discovery and validation groups, respectively. The patients in the discovery group were prospectively enrolled in Guangdong Provincial People’s Hospital from January 2010 to December 2013 on the basis of the same inclusion and exclusion criteria and followed up for all-cause death and major adverse cardiovascular events for up to 5 years. The patients in the validation group were recruited from three hospitals between September 2017 and October 2018 and followed up until December 2019. Baseline information, including demographics, family history, smoking history, medical history, biochemical measurements and medication, were obtained from the hospital information database. This study was approved by the Medical Ethical Review Committee of Guangdong Provincial People’s Hospital (No. GDREC2010137 and GDREC2017071H) and conducted in accordance with the Declaration of Helsinki. Informed consent (No. 20100910, 20170211) was obtained from all individual participants included in the study.

**Sample collection and study endpoints**

Each eligible patient fasted for at least 8 h to minimise the influence of nutrition on metabolite levels. Blood samples were collected in ethylenediaminetetraacetic acid-coated tubes. Whole blood samples were separated into plasma and haemocytes within 2 h via centrifugation at 1000 g for 10 min at 4 °C and then stored at −80 °C for further analysis.

The primary endpoint of interest was all-cause death and major adverse cardiovascular events (MACE). MACE was defined as the occurrence of cardiac death, nonfatal myocardial infarctions, coronary revascularisation and cerebral infection. At each follow-up assessment (every 6 months), the participants were queried about their drinking and smoking history, medication history and onset of new adverse cardiovascular events. Baseline risk factors and medication use were recorded for the enrolled patients. A total of 83 all-cause deaths and 224 MACE were recorded in the two groups with a median of three-year follow up.

**Widely targeted metabolomic profiling**

Widely targeted metabolomic profiling was conducted in the plasma sample of the discovery phase at March 2017, and validation phase in Wuhan Metware Biotechnology at May 2019.

For sample extraction of the discovery phase, plasma was thawed at 4 °C and then vortexed for 10 s. Then, 50 µL of plasma was transferred to 150 µL of pre-cooled methanol to precipitate proteins, vortexed for 3 min at room temperature and then centrifuged at 12,000 rpm for 10 min at 4 °C. Thereafter, the supernatant was centrifuged again at 12,000 rpm for 3 min at 4 °C. Finally, an aliquot of the resulting supernatant was used for metabolomic analysis. Widely targeted metabolomic profiling was conducted on a LC–ESI-MS/MS system (UPLC, Shim-pack UFLC SHIMADZU CBM30A; MS, Applied Biosystems 4500 QTRAP). In total, 202 plasma endogenous metabolites were annotated, mainly including nucleosides, amino acids and derivatives, organ acids and derivatives, hormones, carbohydrates and lipids.

In the validation phase, plasma was thawed on ice, and 150 µL of ice-cold methanol was added to 50 µL of plasma. The mixture was vortexed for 3 min and then centrifuged at 12,000 rpm for 10 min at 4 °C. The supernatant was collected and then centrifuged at 12,000 rpm for 5 min at 4 °C. Finally, the resulting supernatant was used for UPLC-MS/MS analysis. The sample extracts were analyzed using a LC–ESI-MS/MS system (UPLC, Shim-pack UFLC SHIMADZU CBM30A; MS, Applied Biosystems 6500 QTRAP). In total, 600 metabolites were annotated, including 161 identical metabolites detected in the plasma of the discovery phase.

The separation and ESI-Q TRAP-MS/MS detection of the sample from the discovery phase and validation phase was as follows: separation was performed in a Waters ACQUITY UPLC HSS T3 C18 column (pore size 1.8 µM, length 2.1 × 100 mm) by using a gradient solvent system of water (0.04% acetic acid) to acetonitrile (0.04% acetic acid); gradient program, 95:5 V/V at 0 min, 5:95 V/V at 11.0 min, 5:95 V/V at 12.0 min, 95:5 V/V at 12.1 min and 95:5 V/V at 14.0 min; column temperature was held at 40 °C; flow rate was set at 0.35 mL/min and 0.4 mL/min for the samples of the discovery phase and the validation phase, and the injection volumes were 5 μL and 2 μL, respectively.

The effluent was infused to an electrospray ionization (ESI)-triple quadrupole-linear ion trap (QTRAP)–MS equipped with an ESI Turbo Ion-Spray interface, LIT and triple quadrupole (QQQ) scans operated in positive and negative ion modes. The ESI source operation parameters for the discovery phase were as follows: source temperature was held at 550 °C; ion spray voltage (IS) was 5500 V; ion source gas I (GSI), gas II (GSII), curtain gas (CUR) were set at 55, 60, and 25.0 psi, respectively; the collision-activated dissociation (CAD) was high. The ESI source operation parameters for the validation phase were as follows: source temperature was 500 ℃; IS was 5500 V (positive) and -4500 V (negative); and GSI, GSII and CUR were set at 55, 60, and 25.0 psi, respectively; the CAD was high. Instrument tuning and mass calibration were performed with 10 and 100 μmol/L polypropylene glycol solutions in QQQ and LIT modes, respectively. QQQ scans were acquired as MRM experiments with collision gas (nitrogen) set to 5 psi. Individual MRM transitions of declustering potential (DP) and collision energy (CE) were performed with further DP and CE optimization, and each ion pair was scanned and detected on the basis of the optimized DP and CE. At each period, a specific set of MRM transitions was monitored based on the metabolites eluted during this period.

Quality control (QC) samples were pooled with mix plasma from the patients prior to analysis. A QC sample was inserted in every 10 samples under test for the detection duration to monitor repeatability during the analysis. The repeatability of metabolite extraction and detection was judged from the overlapping analysis of total ion flow diagrams (TIC diagrams) between different QC samples. High overlaps of the total ion flow, that is, the retention time and peak strength are consistent, indicating that the signal stability of the mass spectrum is good at different times (Figures S13).

Qualitative analysis of the precursor ion and fragments spectra detected was carried out on the basis of self-built MWDB (metware database) with retention time and ion pairs, as wellas the public database of metabolites information. We used MS/MS spectra to search against public databases to improve confidence in metabolite identification. Metabolite structure resolution is referenced in existing mass spectrometry public databases such as MassBank (http://www.massbank.jp/) [1], HMDB (http://www.hmdb.ca/) [2], METLIN (http://metlin.scripps.edu/index.php) [3]. The metabolite identification was conducted by alignment to the reference standards in our self-built database and public databases, and more information is listed in Table S9. MetaboAnalyst (https://www.metaboanalyst.ca) (version 4.0) [4] and Kyoto Encyclopedia of Genes and Genomes (KEGG) database (http://www.genome.jp/kegg/) [5] was used to analyze the pathway enrichment for the identification of highly enriched metabolic pathways in differential metabolites. The mass spectrum data were processed by Analyst 1.6.3 software (AB Sciex). After obtaining the metabolite spectrum analysis data of different samples, the peak area integral was performed for the mass spectrum peaks, and the integral correction was performed for the mass spectrum peaks of the same metabolite in different samples.

For metabolomic data, raw signals with a coefficient of variation of >50% in the quality control (QC) sample (those with zero ion intensity) were removed. Samples for which metabolites were undetected were imputed with the minimum detected level for the metabolites. Among the 161 annotated metabolites, there were 18 and 13 metabolites with imputed values >10% and >15% in the discovery group respectively, while 10 and 6 metabolites had > 10% and 15% imputed values in the validation group respectively. The metabolites had > 10-15% imputed values are shown in Tables S10-11. An algorithm called Quality Control–Robust Loess Signal Correction (QC–RLSC) was used for correction and integration on seven analytical batches to reduce bias from the batch effect [6]. The use of QC–RLSC is an effective way of normalizing metabolic features to QC samples within an analytical block. The integrated matrix was transformed by Pareto scaling (via mean centring and scaling to the square root of variance) and then exported for further statistical analysis. Given that the value of metabolite ratios may vary widely, the normalisation method used herein involved taking the logarithm (log_10_(A/B)). The correction and integration of analytical batches were performed in statTarget package (version 1.16.1), and Spearman correlation was calculated using the psych package (version 2.0.7) in R (version 3.6.1).

**Genome-wide SNP genotyping and quality control (QC)**

DNA was extracted from the haemocyte samples by using TGuideM16 automatic nucleic acid extractor (Cat. NO. OSE-M16) with the genomic DNA extraction kit (Cat. NO. OSR-M102) of TIANGEN. Concentration quantification and electrophoresis were conducted for DNA QC, and qualified DNA was used for genotyping. The acceptable DNA criteria for genotyping were as follows: (1) The purity of DNA in each sample should be great (OD260/280 ~ 1.8-2.0, OD [optical density]). (2) The concentration of DNA in each sample ≥ 10ng/μl. (3) The total amount of DNA in each sample ≥ 300ng.

The subjects in all groups were genotyped using Global Screening Array (GSA) bead chip. The Illumina Infinium GSA-24 v1.0 bead chip is an advanced genotyping array that provides an economical solution for population-scale genetic studies, variant screening and precision medicine research. Using the iScan System, integrated analysis software, and Infinium high-throughput screening assay, this high-density, 24-sample bead chip provides optimised content for a broad range of applications, delivering the same high-quality, reproducible data that Illumina genotyping arrays have provided for over a decade. The GSA kit includes a convenient package containing bead chips and reagents for amplifying, fragmenting, hybridising, labelling and detecting genetic variants using the high-throughput, streamlined Infinium workflow.

In brief, normalised intensity data were processed using the standard calling algorithm of Illumina. No calls were assigned when the most likely genotype was called beyond the reach of a posterior probability threshold of 0.95 for each individual. Overdispersion biased without calling and/or erroneous genotype assignment visually inspected by intensity cluster plots of significant SNPs showing any of these features were cast off. Finally, 700,078 SNPs were genotyped for the two groups. We combined the genotype data of both groups for subsequent analysis. A systematic QC process was applied to the genotyping data to identify individual outliers and exclude unqualified SNPs. Briefly, the series of filtering standards included the following: (1) maximum per-person missing rate < 0.05; (2) maximum per-SNP missing rate < 0.05; (3) Hardy–Weinberg disequilibrium p-value > 1 × 10^-6^; (4) minor allele frequency (MAF) > 0.05.

**Genotype imputation and post-imputation QC**

Clean genotyping data were prepared and imputed on the basis of 301 Chinese subsets (Chinese Dai in Xishuangbanna, China [CDX] + Han Chinese in Beijing, China [CHB] + Southern Han Chinese [CHS]) in 1000 Human Genomes Project (1000 HGP) phase III haplotype reference panel by using IMPUTE software (version 2) (https://mathgen.stats.ox.ac.uk/impute/impute_v2.html). IMPUTE2 is a genotype imputation and haplotype phasing program based on the work of Howie et al [7]. In brief, genotype imputation is an approach for inferring unmarked genotypes based on phased haplotypes in a dense genotyped reference panel. Genotype imputation can help in the study of additional markers economically and effectively. The imputed data were cleaned with post-imputation QC procedures by removing low-imputation-quality (info < 0.6), low-MAF (< 0.05), and average-certainty best-guess genotypes (< 0.95). After filtering, the data were included in subsequent analyses.

**metaboGWAS and meta-analysis**

The primary association testing was conducted at each SNP (after genotype imputation) for the concentrations of 161 metabolites present in the discovery and validation groups after the QC steps. All association analyses were conducted via PLINK software (version 2.0) [8] by using linear regression models. The first 10 principal component (PCs), sex, age, aspartate aminotransferase (AST), estimated glomerular filtration rate (eGFR), antihypertensive drugs, hypertension and diabetes were included as covariates in both groups. The 10 PCs in this study mean the ten main ancestral PCs from genomic in our data, which could reflect the substructures within the crowd. Previous literature [9] has shown the substructures within the Han Chinese population and the importance of adjustment in association studies.

Inverse-variance weighted (IVW) meta-analysis was based on the effect sizes, and the standard errors adjusting for genomic control were used to combine the association results for the two groups in this study. Cochran’s Q test was used to test the heterogeneity in each association between datasets. The meta-analysis and heterogeneity test above were carried out using METAL software [10]. Significant heterogeneity (P < 0.001) of effects between the two groups and at p-value > 0.001 in either group was removed after meta-analysis. To correct for multiple hypothesis testing, we considered a Bonferroni adjusted p-value of P = 5 × 10^-8^ / 161 = 3.11 × 10^-10^ as a cut-off for genome significance. A lead SNP was defined as the SNP with the lowest p-value against any metabolites at that locus. All associations that passed the cut-off were assigned to independent loci (lead SNPs and SNPs beyond 500 kb and r^2^ < 0.2) for each metabolite, and the assignments were described later for MR study.

**Metabolite ratio analyses**

We tested the association between each SNP with 12,880 (161 × 160/2) ratios of each pair of metabolites for further discovery by using PLINK2 following the same method as that of the primary analysis of single metabolites. The genome significance cut-off for ratio association test was defined as P = 5 × 10^-8^ / 12,880 = 3.88 × 10^-12^. Linkage disequilibrium (LD) analysis was conducted to select the independent loci; the details are presented later. P-gain can be used as a standard to integrate the results of association analysis between single metabolites and ratios, and the systematic definition of p-gain shown in Ref. [11] is widely used in metaboGWAS research. In short, p-gain follows this calculation rule: p-gain = min (P [metabolite A], P [Metabolite B]) / P[A/B]). The threshold of p-gain is based on assumed level of significance of α/B [11]. Given that α = 0.05 is used as a type I error rate, the critical value for p-gain can be described as B/(2×α), i.e., 10⋅B [12]. The uncorrected critical value of 10 was multiplied by the number of test B for Bonferroni correction. Thus, the threshold of p-gain was set as 10 × 161 × 12 = 19,320 (10 × number of metabolites × number of independent SNP obtained from single metabolites). The following rules apply in the selection between the ratio and a single metabolite in the association results after significant screening. Evidence for ratios was reported only when statistical evidence for the ratio was stronger than that for a metabolite alone [13]. Ratios were considered only if their p-gain was > 19,320, following the previously presented formalisation. (1) If a locus was associated more strongly with a ratio than with a metabolite (as indicated by p-gain > 19,320), then the metabolite and the best ratio of all significant ratios were reported; (2) if a locus was associated with a metabolite and one or more ratios at genome-wide significance, but p-gain < 19,320, then only the association with the metabolite was reported; (3) if a locus was associated only with one or more ratios, then the ratios were reported. In addition, if the numerator or denominator in the ratio was one and only one in a single metabolite, then such ratio was not reported.

**Fine mapping of metaboQTLs**

To refine further the identity of metabolite-associated variants, we performed LD analyses by using PLINK software. The lead SNPs from the analysis of single metabolites and ratios were pooled. Lead SNP was defined as the SNP with the most significant p-value, and LD clumping was conducted. Other significant SNPs were compared with the lead SNPs; those that met the following three conditions at the same time were considered as linked sites, and only the lead SNP was retained in the interlinked sites: (1) located on the same chromosome; (2) with distance from each other less than 500 kb; (3) r^2^ > 0.2. The final set of SNPs was annotated with ANNOVAR software [14], and the genes nearest to the lead SNP were obtained. Eventually, we searched the GWAS Catalog (<https://www.ebi.ac.uk/gwas/>) [15] (last data release on 2020-08-13) for the reported or possibly novel loci.

**Biological annotation**

Several databases were used for biological annotation; they included GTEx portal [16] (version 8, www.gtexportal.org), Online Mendelian Inheritance in Man database [17] (www.omim.org), CHEMBL targets database [18] (www.ebi.ac.uk/chembl), Human Metabolome Database [19] (https://hmdb.ca/) and PubChem [20] (https://pubchem.ncbi.nlm.nih.gov/).

**Association between metaboQTL-related metabolites and endpoint events**

Cox regression analysis was performed to identify the relationship between metabolites that were associated with the metaboQTLs and the risk of death and MACE and to estimate hazard ratio (HR) and 95% confidence intervals (CIs), adjusting for sex, age, AST, eGFR, hypertension and diabetes, cardiovascular family history and smoking history. Given that the follow-up period in validation group is short (at maximum ~2 years), Cox regression was only performed in the discovery group (the follow-up period ~5 years). We also performed Cox regression analysis on genotype data with endpoints in the discovery group to identify the loci that were associated with the risk of death and MACE and to estimate the beta value and standard error for later Mendelian randomisation (MR) study. Cox regression for metabolite endpoints was conducted using survival package (version 3.2-3) in R, and SNP endpoints were determined using the SurvivalGWAS_SV software (version 1.3.2) [21].

**MR study**

For metabolite associated with death or MACE risk, we selected the related SNPs that showed association at P < 1 × 10^-5^. A relaxed statistical threshold can explain the large variation when limited genome-wide significant SNPs were available for exposures in the MR study [22]. LD analysis was conducted to retain the SNPs with the lowest p-value and the independent SNPs with them (r^2^ < 0.001 in a 10,000 kb window or two SNPs beyond 10,000 kb) as the independent instruments. We perform both one-sample and two-sample MR approaches to estimate the causal relationship and the steps are as follows:

First, we used two-sample MR approach to estimate the causal effect of exposure on outcome. Exposure association results come from the validation group, and the outcome association results come from the discovery group. The inverse variance weighted (IVW) method was used as a primary test to estimate the causal effect for two-sample MR analysis. Given that biased estimates in the presence of horizontal pleiotropy can be yielded by the IVW approach [23], we used four additional MR models, including MR Egger regression, the weighted median estimator and the simple and weighted mode-based estimators, to estimate the causal relationship between exposure and outcome. Furthermore, we performed sensitivity analyses, including heterogeneity test, pleiotropy test, and leave-one-out analyses. The two-sample MR analyses were conducted using TwoSampleMR package [24] (version 0.4.22) in R.

Second, considering that one-sample MR approach could provide a stronger causal test ability than two-sample MR approach, so we used the two-stage least squares (2SLS) method to perform the one-sample MR analysis in the discovery group. In the first-stage, the exposure was regressed on the independent genetic instrument and the predicted values of exposure are taken from the regression model. In the second stage, the outcome is regressed over the predicted values of exposure by using Cox regression in R.

**Supplemental Table Legends**

**Table S1** Spearman correlation coefficient of metabolites in the discovery group.

**Table S2** P-value of Spearman correlation analysis of metabolites in the discovery group.

**Table S3** Summary data of two groups.

**Table S4** Study-specific association statistics.

**Table S5** Biological annotation for metabolite quantitative trait loci (metaboQTLs).

**Table S6** The effects of metaboQTL-related metabolites on clinical endpoints in the discovery group.

**Table S7** Five MR models for assocaitions between 3-indolepropionic acid and MACE.

**Table S8** The results of sensitivity analyses between 3-indolepropionic acid and MACE in MR.

**Table S9** The ionisation modes and ion pairs of the metabolites.

**Table S10** Eighteen metabolites had > 10% imputed values in the discovery group.

**Table S11** Ten metabolites had > 10% imputed values in the validation group.

**Supplemental Figure Legends**

**Figure S1** Flow chart of the enrolment of the CAD patients.

**Figure S2** Q-Q plot of the ratio of L-histidine and uric acid p-values in meta-analysis.

**Figure S3** Q-Q plot of glycocholic acid p-values in meta-analysis.

**Figure S4** Q-Q plot of glycochenodeoxycholic acid p-values in meta-analysis.

**Figure S5** Q-Q plot of inosine p-values in meta-analysis.

**Figure S6** Q-Q plot of 3-Indolepropionic acid p-values in meta-analysis.

**Figure S7** Q-Q plot of 2-Methylsuccinic acid p-values in meta-analysis.

**Figure S8** Q-Q plot of dodecanedioic aicd p-values in meta-analysis.

**Figure S9** Q-Q plot of the ratio of cis-5,8,11,14,17-Eicosapentaenoic Acid (C20:5n3) and LysoPC 20:2 p-values in meta-analysis.

**Figure S10** Q-Q plot of eudesmic acid p-values in meta-analysis.

**Figure S11** Q-Q plot of 1,4-dihydro-1-Methyl-4-oxo-3-pyridinecarboxamide p-values in meta-analysis.

**Figure S12** Q-Q plot of hypoxanthine p-values in meta-analysis.

**Figure S13** Representative total ion flow diagrams between different QC samples under positive ion mode (a) and negative ion mode (b). Highly overlapping of the total ion chromatogram (TIC) of different QC samples for mass spectrometric detection and analysis showed the repeatability of the extraction and detection.

**Figure S14** Scatter plot for the genetic associations of 3-indolepropionic acid on the risk of MACE. Four two-sample MR models (inverse variance weighted, weighted median estimator, simple mode-based estimator, and weighted mode-based estimator) show the positive effect value, which were opposite to the Cox regression model result.

**Supplemental Figures**

**Figure S1**

**
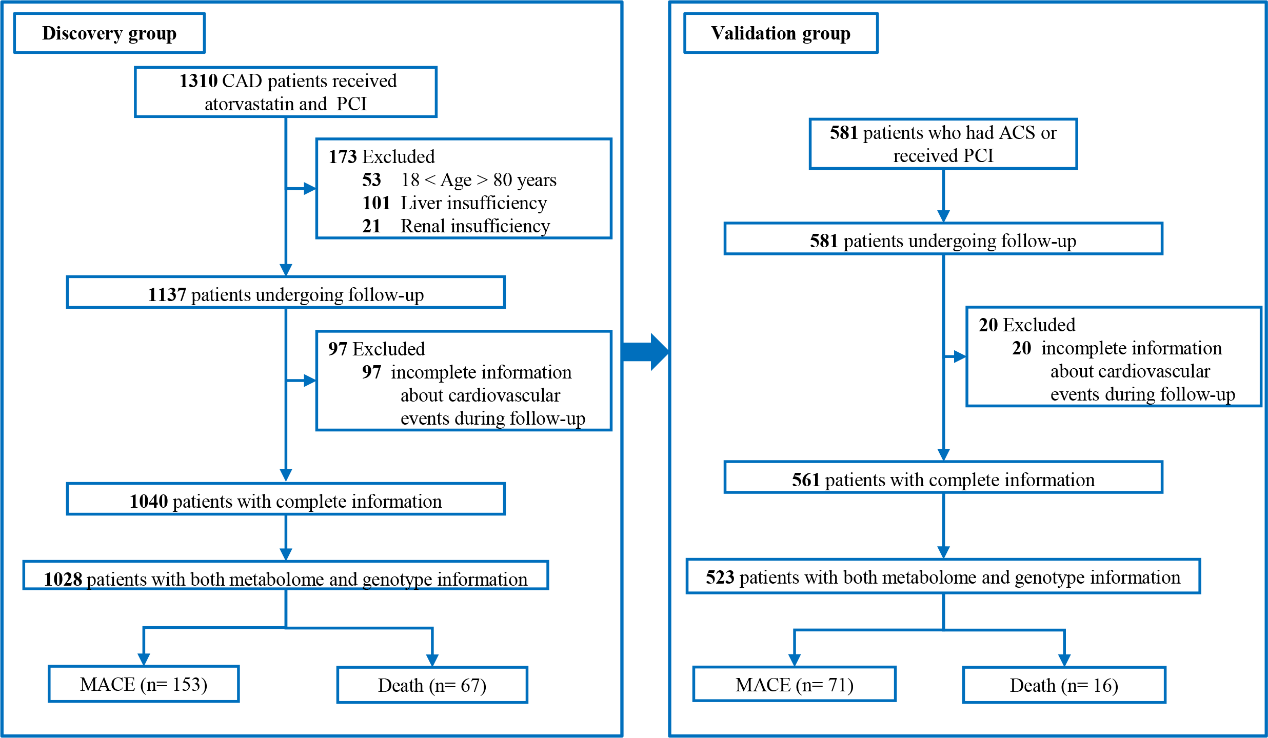
**

**Figure S2**


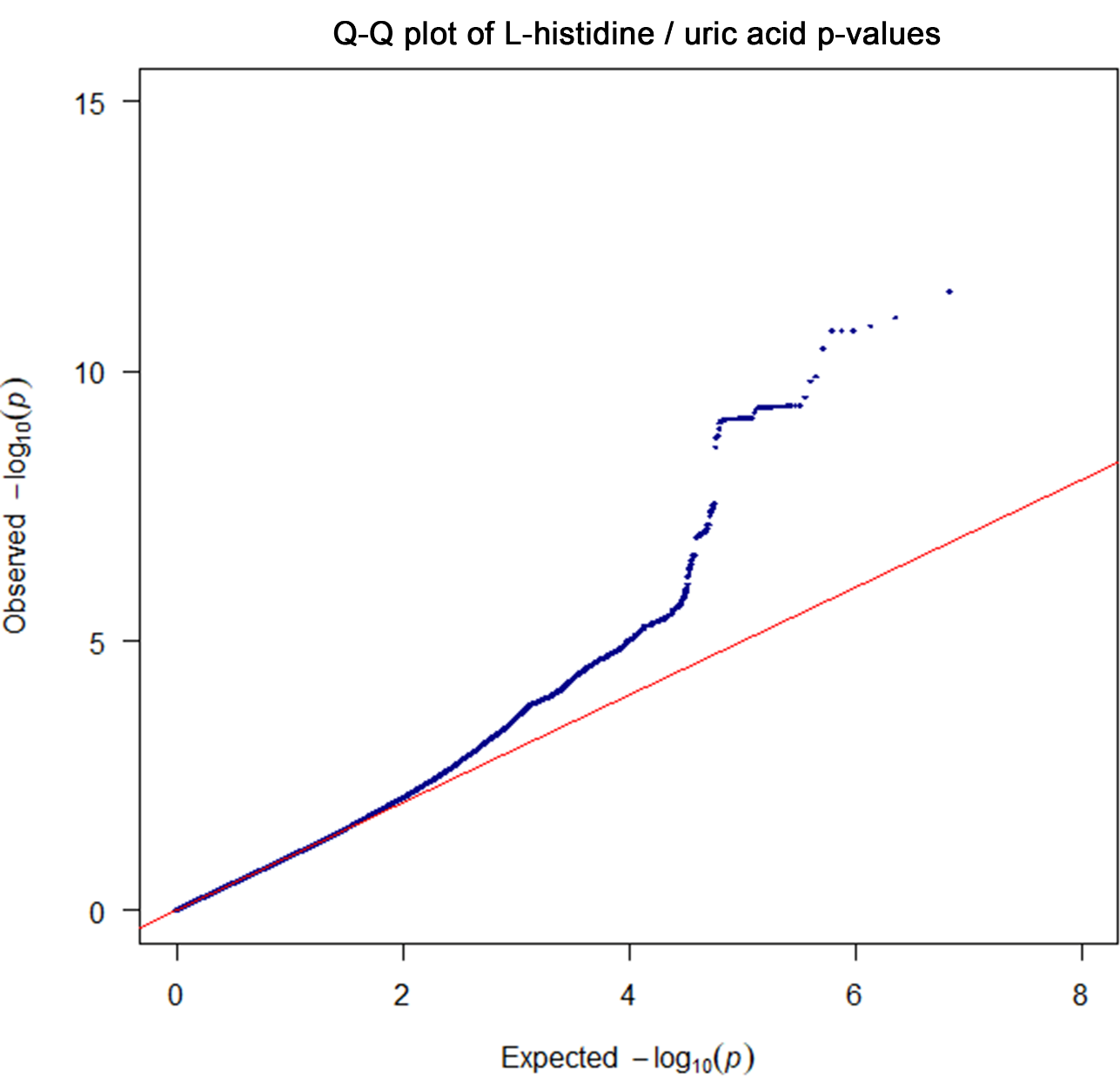


**Figure S3**


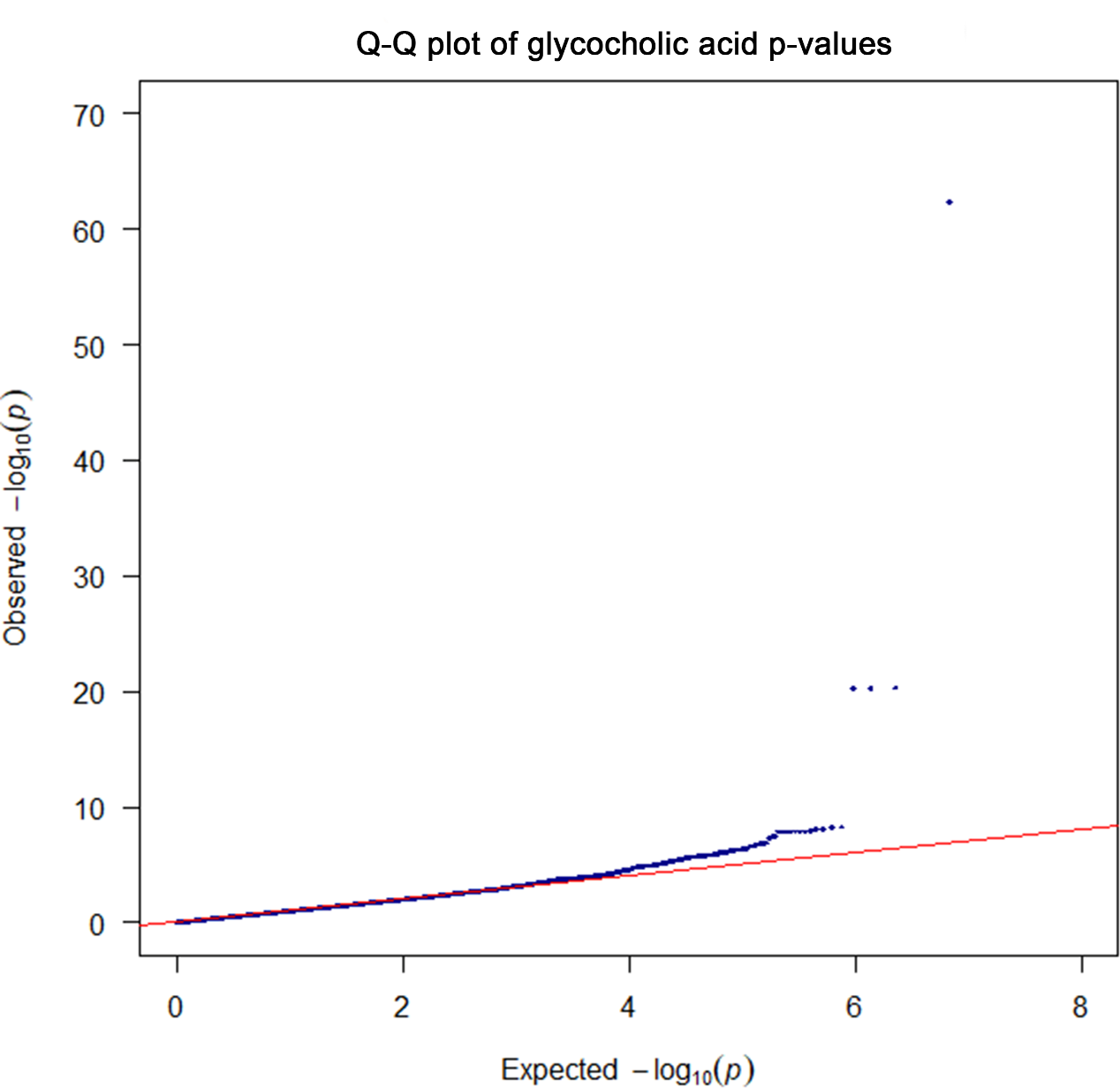


**Figure S4**


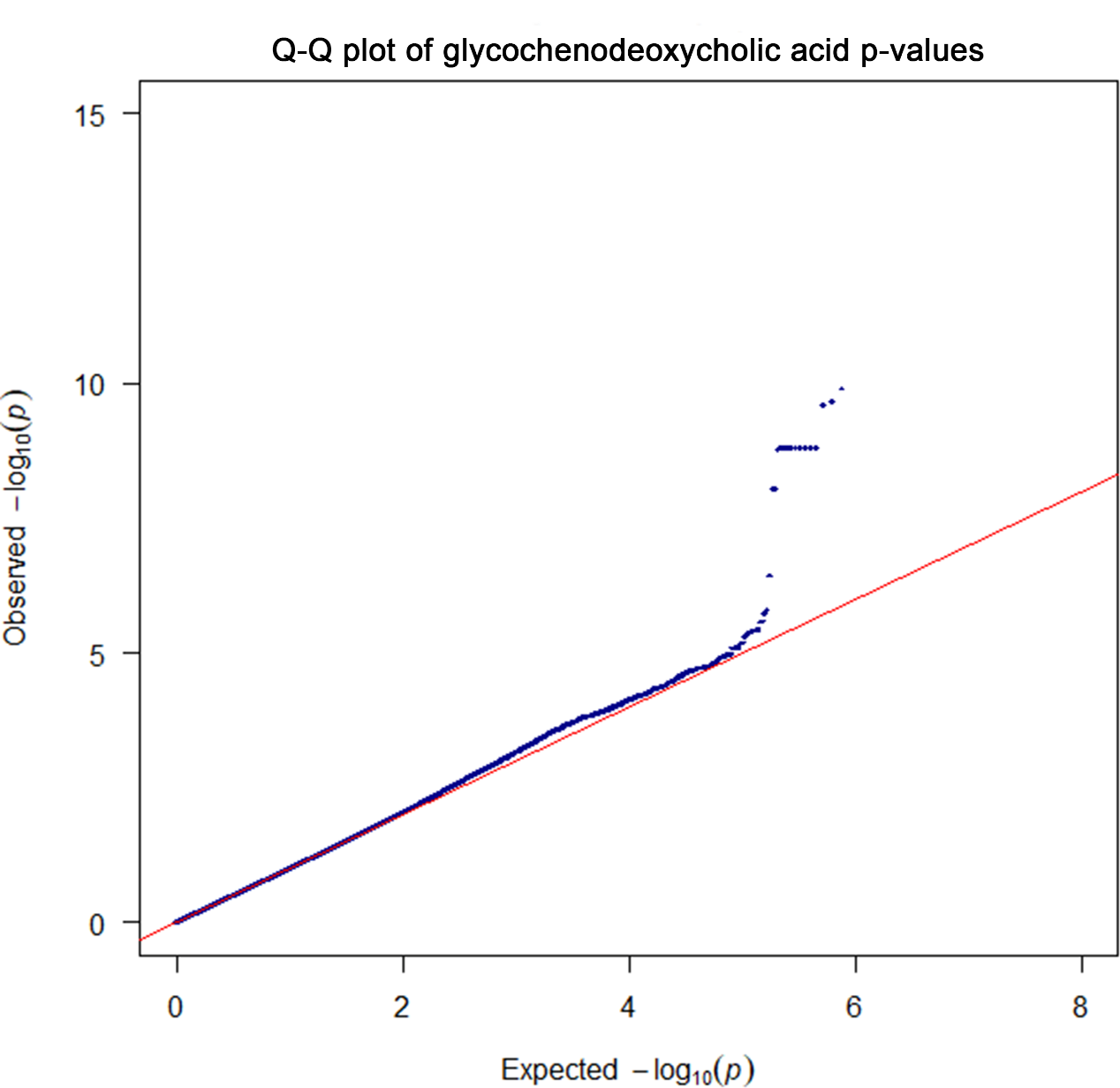


**Figure S5**


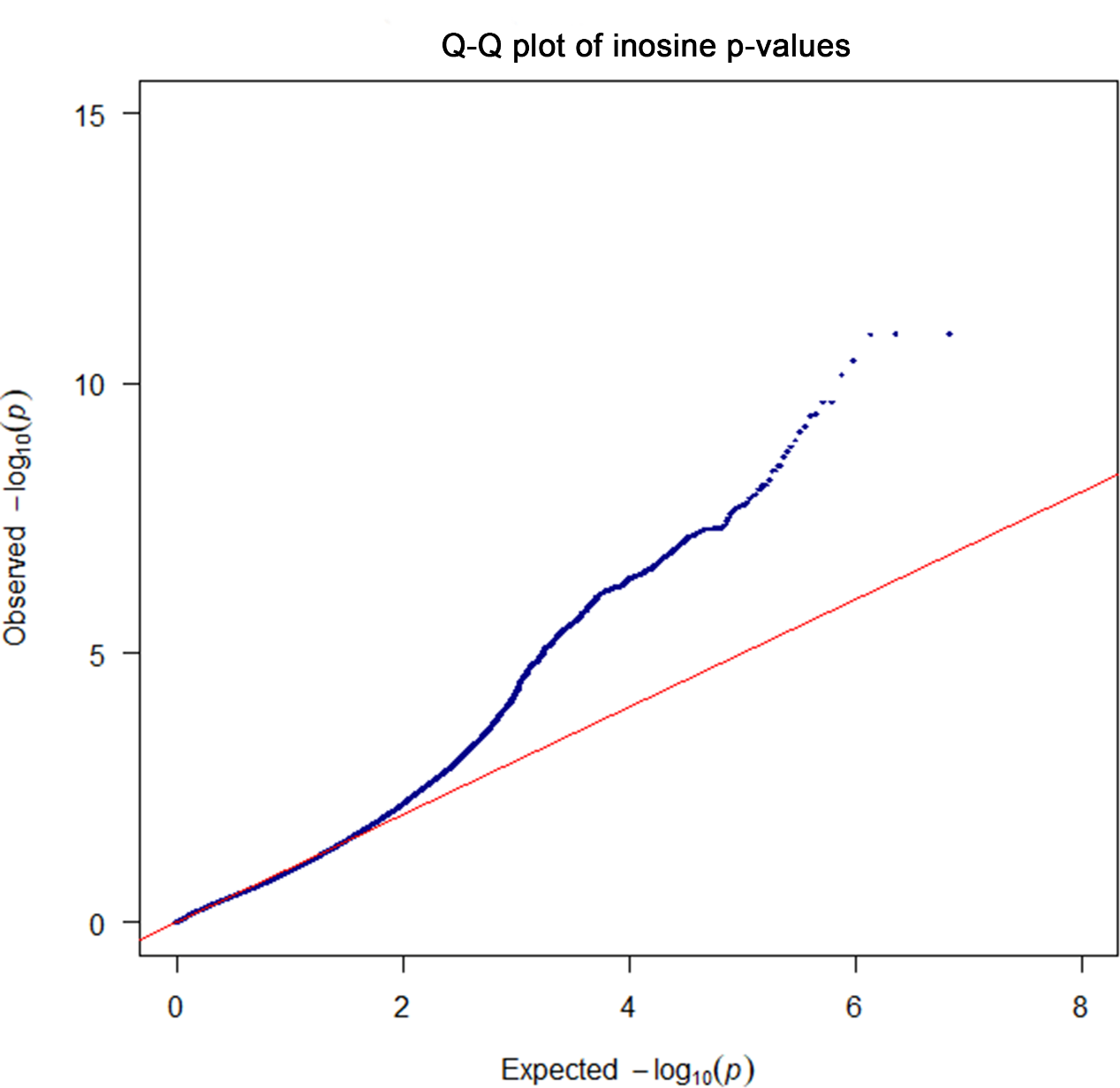


**Figure S6**


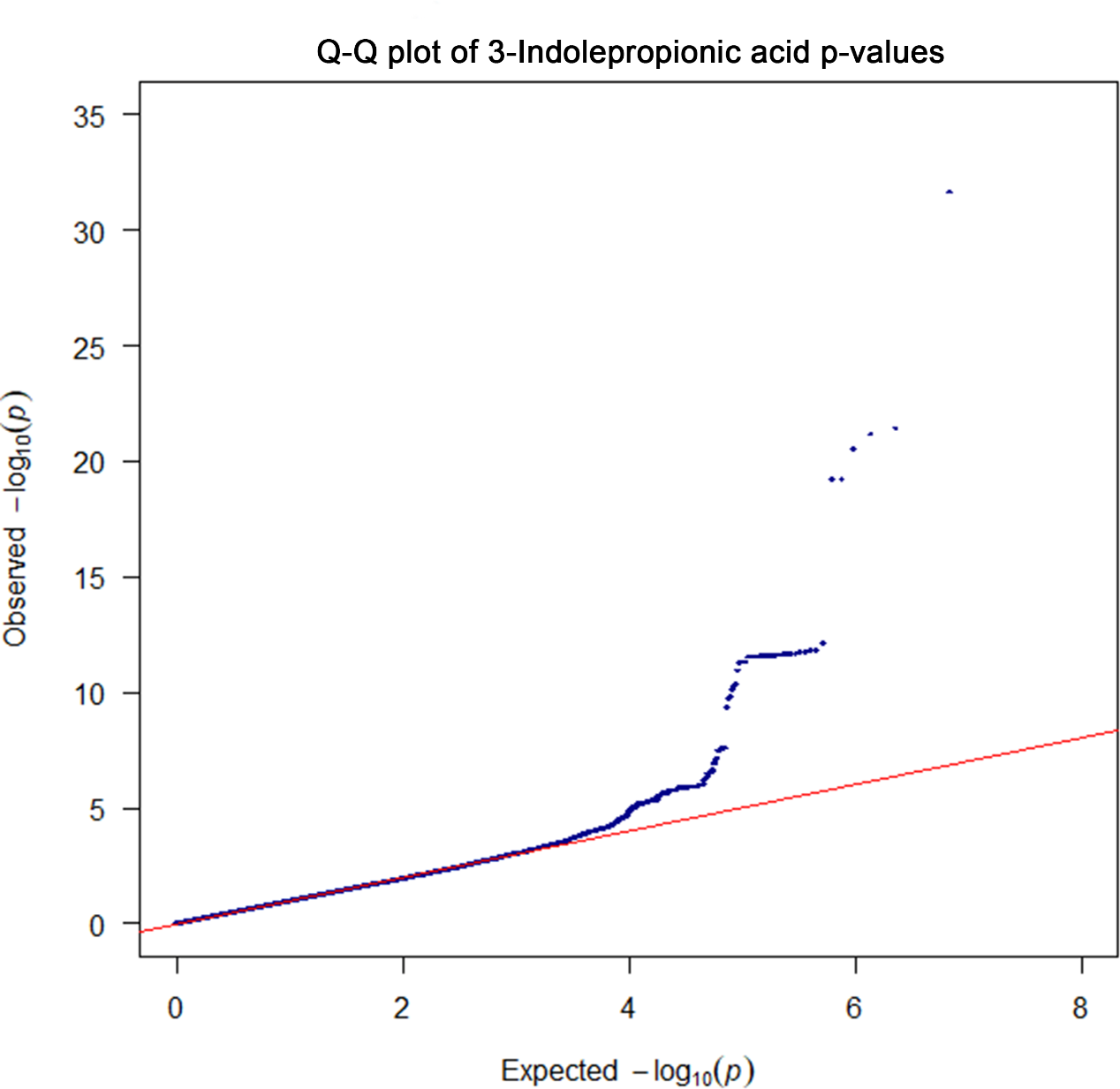


**Figure S7**


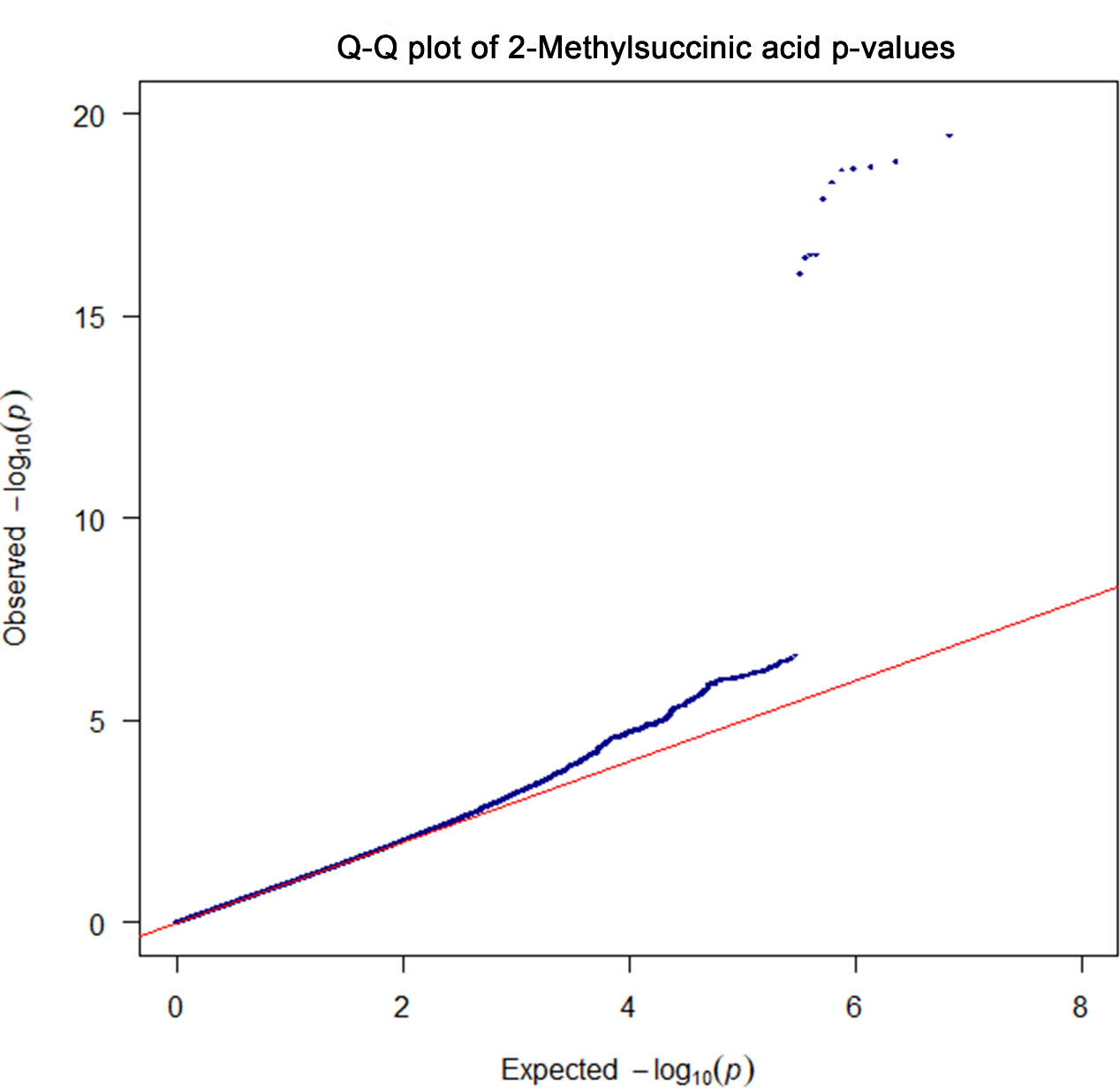


**Figure S8**


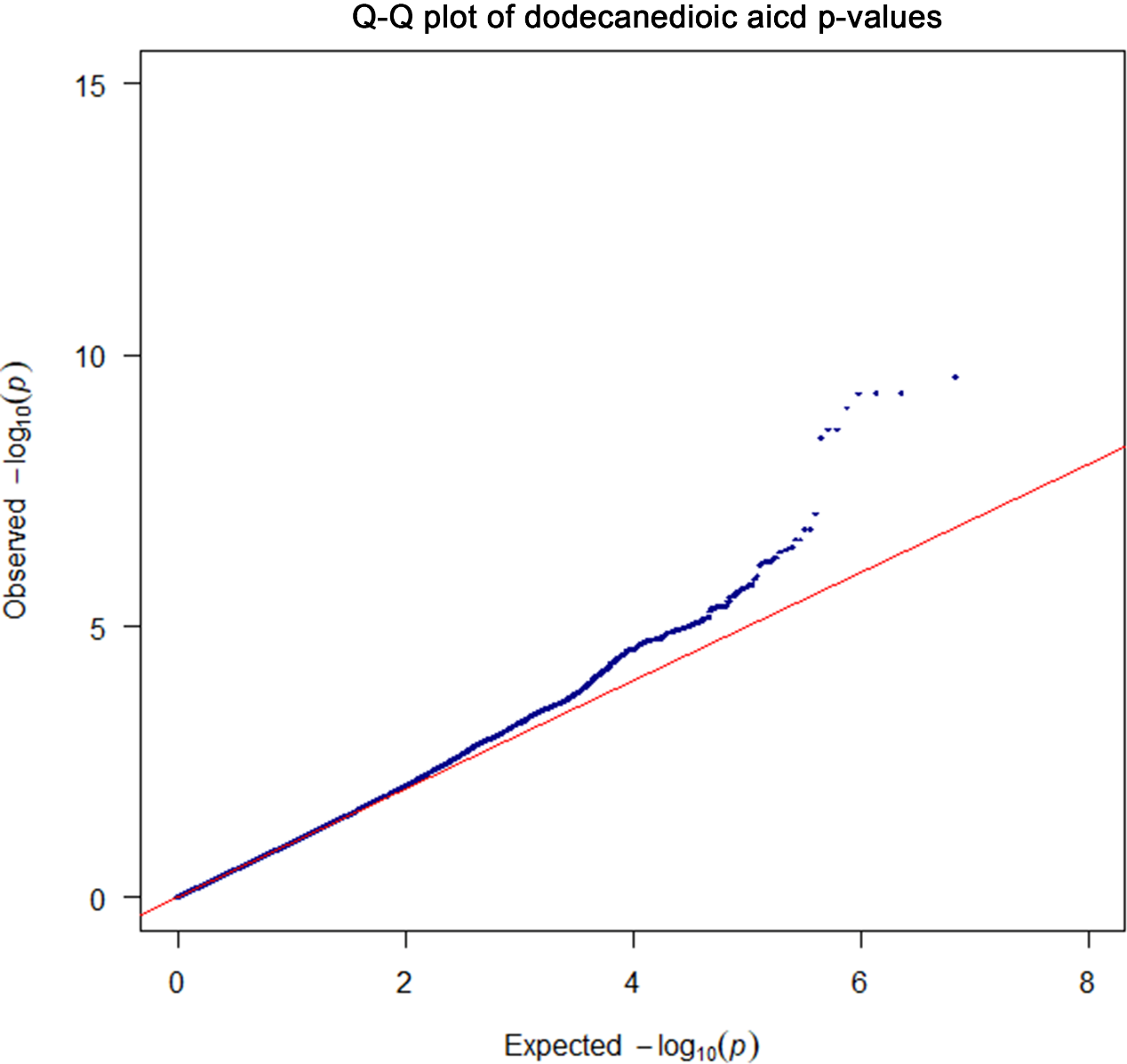


**Figure S9**


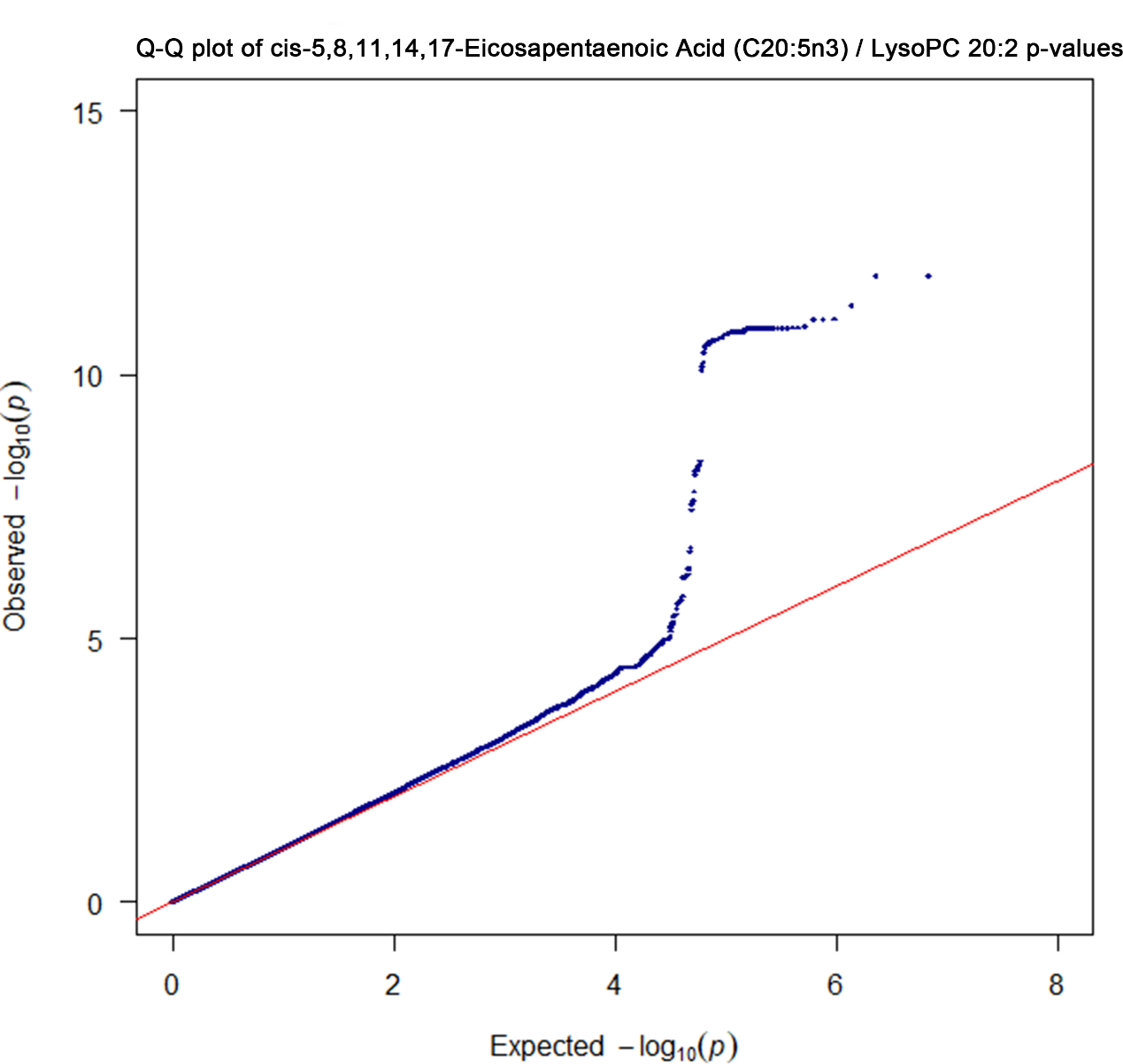


**Figure S10**


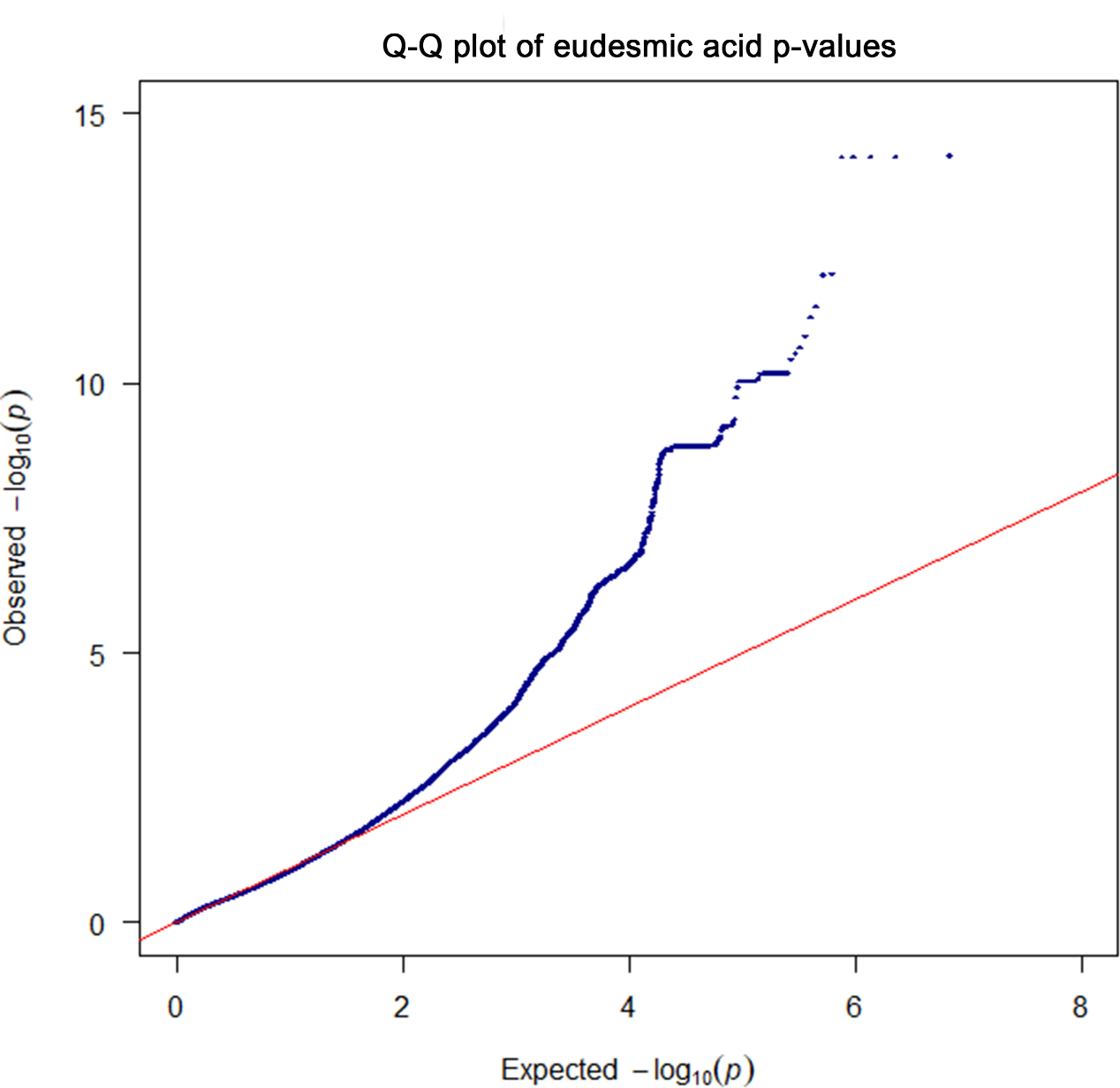


**Figure S11**


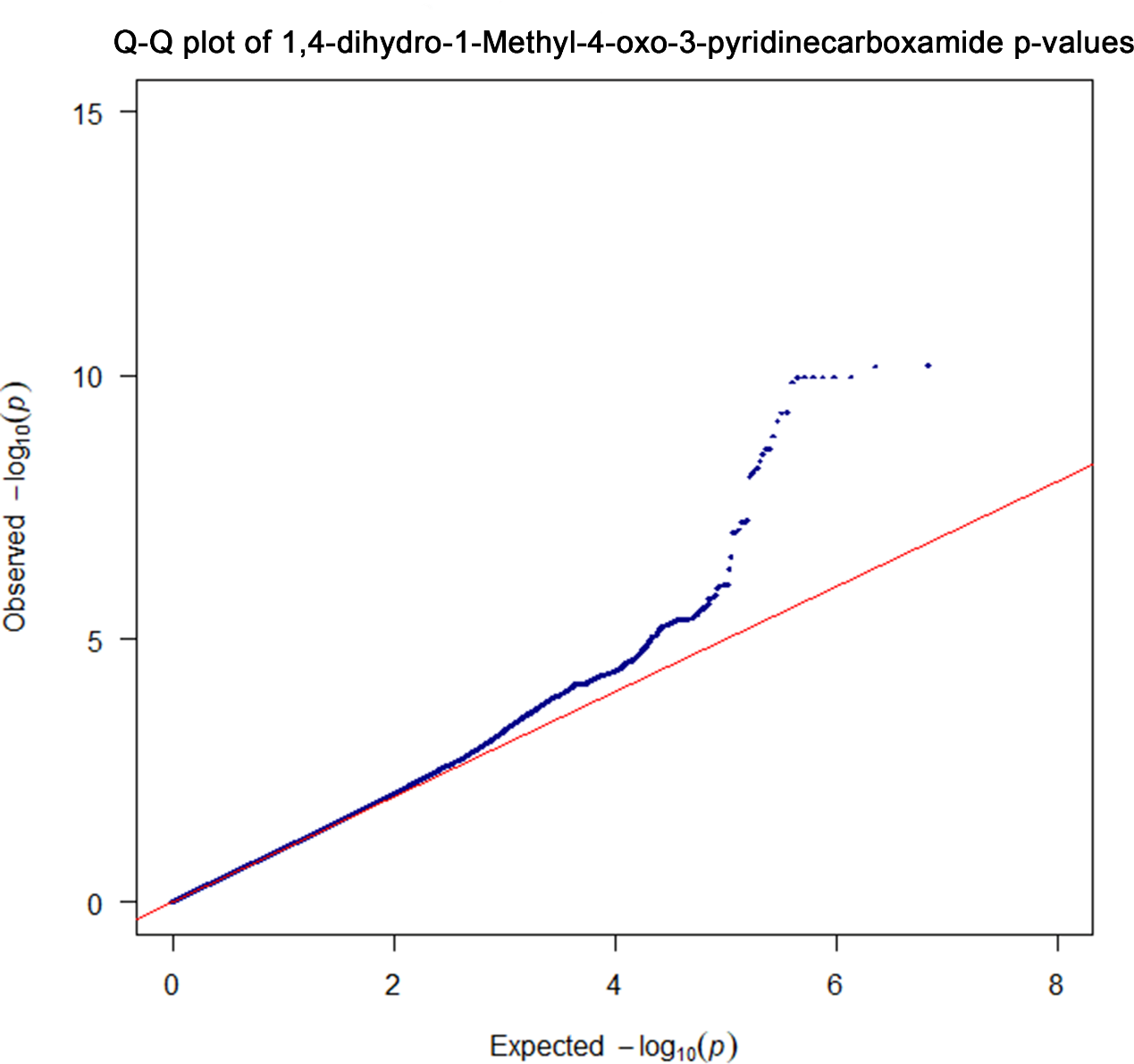


**Figure S12**


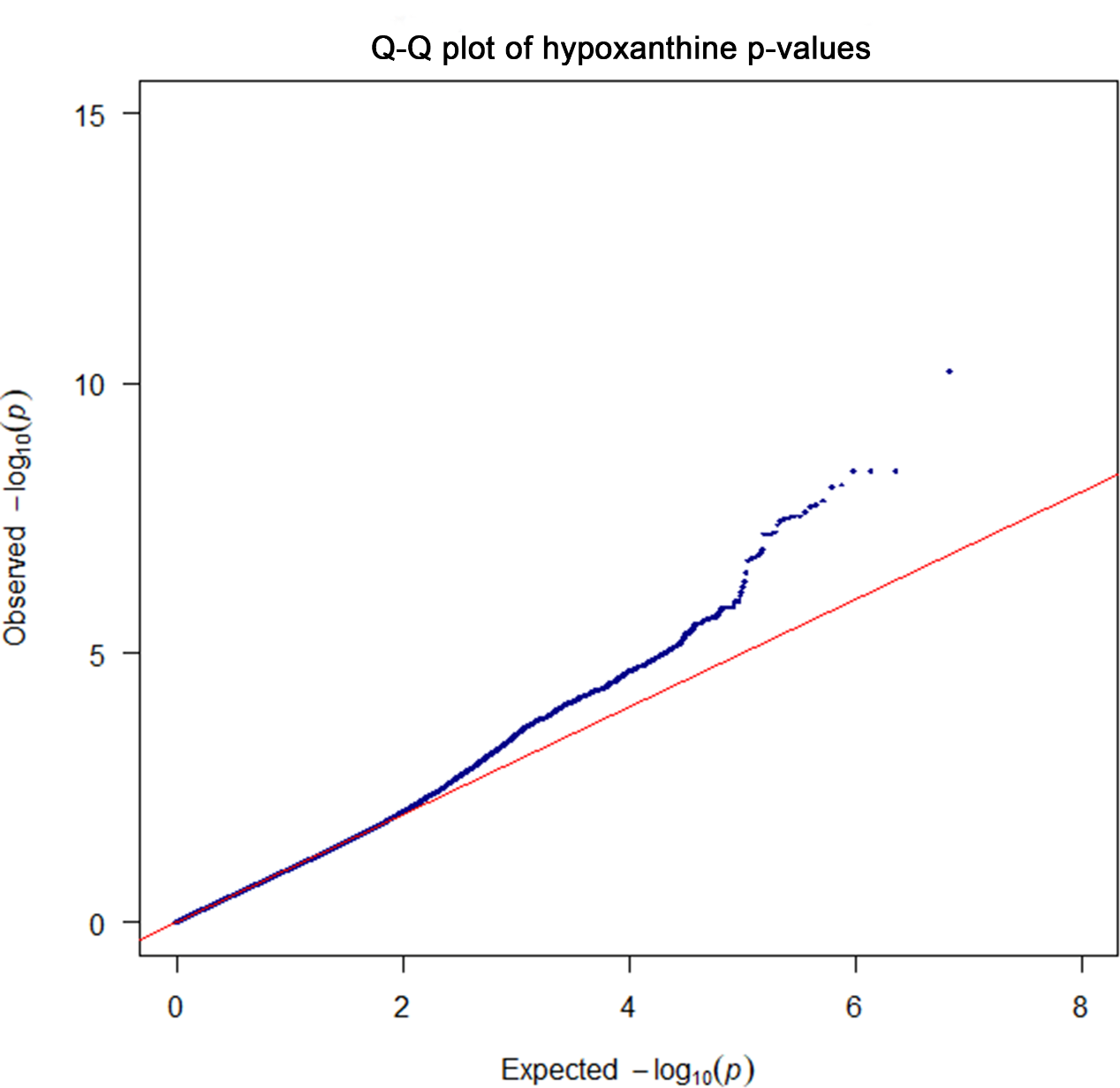


**Figure S13**


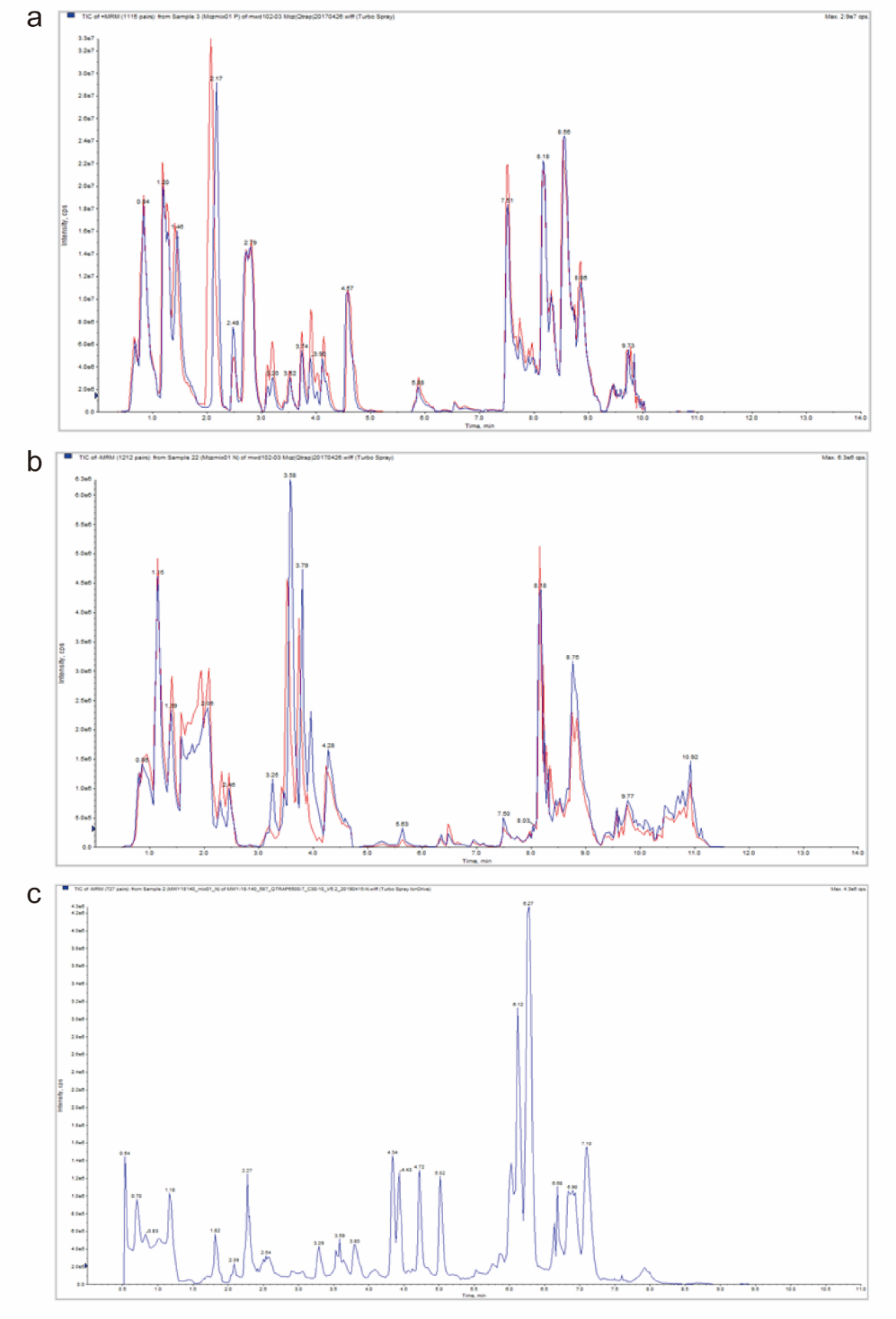


**Figure S14**


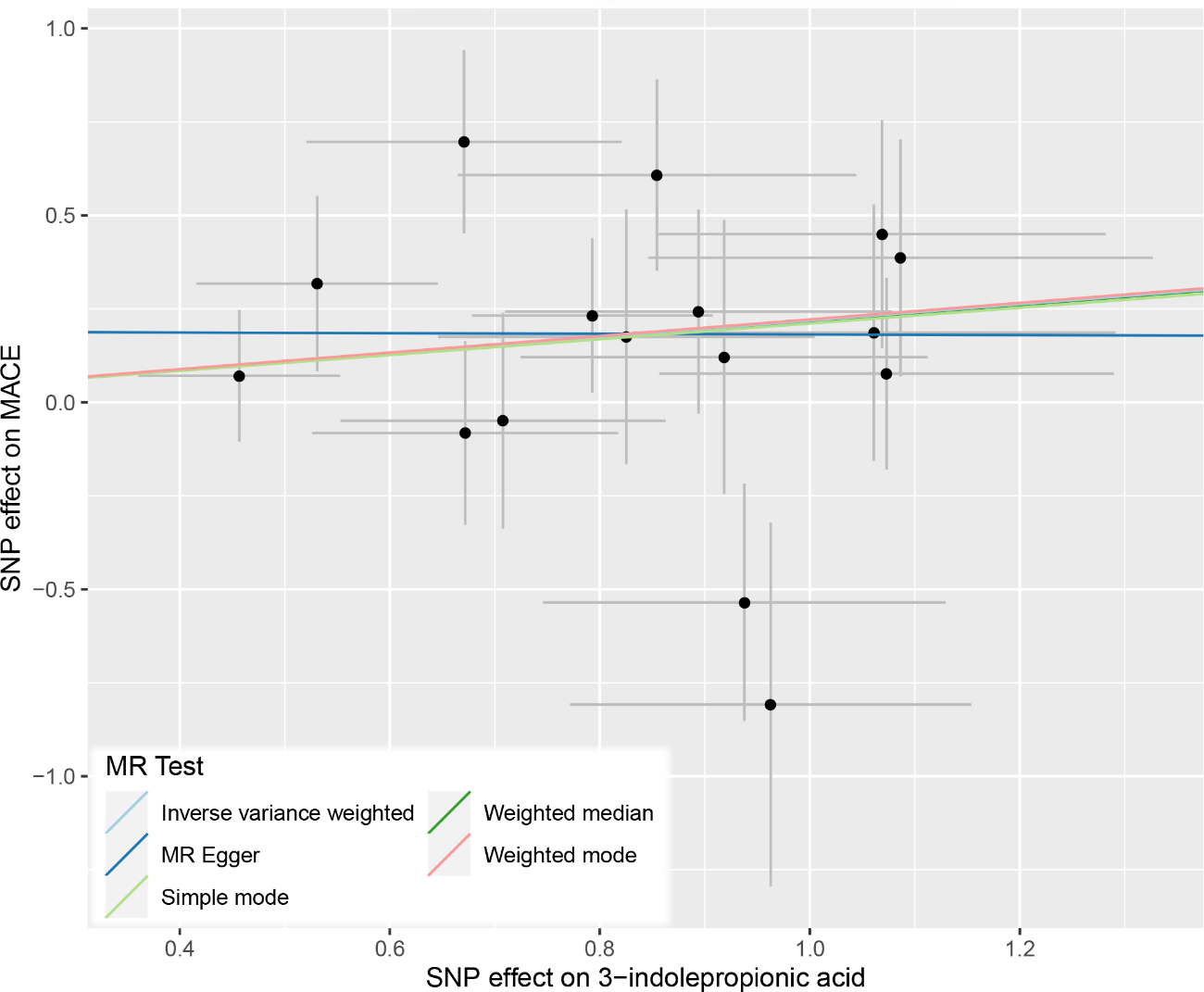


**Reference**

1. Horai H, Arita M, Kanaya S, et al. MassBank: a public repository for sharing mass spectral data for life sciences. Journal of mass spectrometry : JMS. 2010; 45: 703-14.

2. Wishart DS, Feunang YD, Marcu A, et al. HMDB 4.0: the human metabolome database for 2018. Nucleic acids research. 2018; 46: D608-d17.

3. Smith CA, O'Maille G, Want EJ, et al. METLIN: a metabolite mass spectral database. Therapeutic drug monitoring. 2005; 27: 747-51.

4. Xia J, Sinelnikov IV, Han B, et al. MetaboAnalyst 3.0--making metabolomics more meaningful. Nucleic acids research. 2015; 43: W251-7.

5. Kanehisa M, Goto S. KEGG: kyoto encyclopedia of genes and genomes. Nucleic acids research. 2000; 28: 27-30.

6. Luan H, Ji F, Chen Y, et al. statTarget: A streamlined tool for signal drift correction and interpretations of quantitative mass spectrometry-based omics data. Analytica chimica acta. 2018; 1036: 66-72.

7. Howie BN, Donnelly P, Marchini J. A flexible and accurate genotype imputation method for the next generation of genome-wide association studies. PLoS genetics. 2009; 5: e1000529.

8. Chang CC, Chow CC, Tellier LC, et al. Second-generation PLINK: rising to the challenge of larger and richer datasets. GigaScience. 2015; 4: 7.

9. Chen J, Zheng H, Bei JX, et al. Genetic structure of the Han Chinese population revealed by genome-wide SNP variation. American journal of human genetics. 2009; 85: 775-85.

10. Willer CJ, Li Y, Abecasis GR. METAL: fast and efficient meta-analysis of genomewide association scans. Bioinformatics (Oxford, England). 2010; 26: 2190-1.

11. Petersen AK, Krumsiek J, Wägele B, et al. On the hypothesis-free testing of metabolite ratios in genome-wide and metabolome-wide association studies. BMC bioinformatics. 2012; 13: 120.

12. Yousri NA, Fakhro KA, Robay A, et al. Whole-exome sequencing identifies common and rare variant metabolic QTLs in a Middle Eastern population. Nature communications. 2018; 9: 333.

13. Shin SY, Fauman EB, Petersen AK, et al. An atlas of genetic influences on human blood metabolites. Nature genetics. 2014; 46: 543-50.

14. Wang K, Li M, Hakonarson H. ANNOVAR: functional annotation of genetic variants from high-throughput sequencing data. Nucleic acids research. 2010; 38: e164.

15. Welter D, MacArthur J, Morales J, et al. The NHGRI GWAS Catalog, a curated resource of SNP-trait associations. Nucleic acids research. 2014; 42: D1001-6.

16. The Genotype-Tissue Expression (GTEx) project. Nature genetics. 2013; 45: 580-5.

17. Hamosh A, Scott AF, Amberger JS, et al. Online Mendelian Inheritance in Man (OMIM), a knowledgebase of human genes and genetic disorders. Nucleic acids research. 2005; 33: D514-7.

18. Gaulton A, Bellis LJ, Bento AP, et al. ChEMBL: a large-scale bioactivity database for drug discovery. Nucleic acids research. 2012; 40: D1100-7.

19. Wishart DS, Tzur D, Knox C, et al. HMDB: the Human Metabolome Database. Nucleic acids research. 2007; 35: D521-6.

20. Kim S, Thiessen PA, Bolton EE, et al. PubChem Substance and Compound databases. Nucleic acids research. 2016; 44: D1202-13.

21. Syed H, Jorgensen AL, Morris AP. SurvivalGWAS_SV: software for the analysis of genome-wide association studies of imputed genotypes with "time-to-event" outcomes. BMC bioinformatics. 2017; 18: 265.

22. Yang J, Yan B, Zhao B, et al. Assessing the Causal Effects of Human Serum Metabolites on 5 Major Psychiatric Disorders. Schizophrenia bulletin. 2020; 46: 804-13.

23. Hwang LD, Lawlor DA, Freathy RM, et al. Using a two-sample Mendelian randomization design to investigate a possible causal effect of maternal lipid concentrations on offspring birth weight. International journal of epidemiology. 2019; 48: 1457-67.

24. Hemani G, Zheng J, Elsworth B, et al. The MR-Base platform supports systematic causal inference across the human phenome. eLife. 2018; 7.
